# Supplementary material for: FastqPuri: high-performance preprocessing of RNA-seq data
Source: BMC Bioinformatics. 2019 May 3;20:226. doi: 10.1186/s12859-019-2799-0 (PMC6500068; doi:10.1186/s12859-019-2799-0)
Supplement: Supplementary file 2 — Archive of FastqPuri. Archive containing all files needed to install and run FastqPuri v1.0.6. Date stamp March 22, 2019. (GZ 47,819 kb) [file 12859_2019_2799_MOESM2_ESM.gz › FastqPuri-1.0.6/html/fq__read_8h_source.html]

FastqPuri: include/fq\_read.h Source File


FastqPuri


- include

fq\_read.h

Go to the documentation of this file.

1 /\*\*\*\*\*\*\*\*\*\*\*\*\*\*\*\*\*\*\*\*\*\*\*\*\*\*\*\*\*\*\*\*\*\*\*\*\*\*\*\*\*\*\*\*\*\*\*\*\*\*\*\*\*\*\*\*\*\*\*\*\*\*\*\*\*\*\*\*\*\*\*\*\*\*\*\*

2  \* Copyright (C) 2017 by Paula Perez Rubio \*

3  \* \*

4  \* This file is part of FastqPuri. \*

5  \* \*

6  \* FastqPuri is free software: you can redistribute it and/or modify \*

7  \* it under the terms of the GNU General Public License as \*

8  \* published by the Free Software Foundation, either version 3 of the \*

9  \* License, or (at your option) any later version. \*

10  \* \*

11  \* FastqPuri is distributed in the hope that it will be useful, \*

12  \* but WITHOUT ANY WARRANTY; without even the implied warranty of \*

13  \* MERCHANTABILITY or FITNESS FOR A PARTICULAR PURPOSE. See the \*

14  \* GNU General Public License for more details. \*

15  \* \*

16  \* You should have received a copy of the GNU General Public License \*

17  \* along with FastqPuri. \*

18  \* If not, see <http://www.gnu.org/licenses/>. \*

19  \*\*\*\*\*\*\*\*\*\*\*\*\*\*\*\*\*\*\*\*\*\*\*\*\*\*\*\*\*\*\*\*\*\*\*\*\*\*\*\*\*\*\*\*\*\*\*\*\*\*\*\*\*\*\*\*\*\*\*\*\*\*\*\*\*\*\*\*\*\*\*\*\*\*\*\*/

20

29 #ifndef FQ\_READ\_H\_

30 #define FQ\_READ\_H\_

31

32 #include "config.h"

33

37 typedef struct \_fq\_read {

38  char line1[READ\_MAXLEN];

39  char line2[READ\_MAXLEN];

40  char line3[READ\_MAXLEN];

41  char line4[READ\_MAXLEN];

42  int L;

43  int start;

45  int Lhalf;

46  char extended[READ\_MAXLEN];

47  unsigned char pack[(READ\_MAXLEN+1)/2];

48  unsigned char packsh[(READ\_MAXLEN+1)/2];

49  int L\_ad;

50  int L\_ext;

51  int L\_pack;

52  int L\_packsh;

53 } Fq\_read;

54

55 int get\_fqread(Fq\_read\* seq, char\* buffer, int pos1, int pos2,

56  int nline, int read\_len, int filter);

57

58 int string\_seq(Fq\_read \*seq, char \*char\_seq);

59

60 #endif // endif FQ\_READ\_H\_

\_fq\_read::L

int L

**Definition:** fq\_read.h:42

\_fq\_read::line3

char line3[READ\_MAXLEN]

**Definition:** fq\_read.h:40

\_fq\_read::line2

char line2[READ\_MAXLEN]

**Definition:** fq\_read.h:39

\_fq\_read::line4

char line4[READ\_MAXLEN]

**Definition:** fq\_read.h:41

\_fq\_read::L\_ext

int L\_ext

**Definition:** fq\_read.h:50

\_fq\_read::L\_ad

int L\_ad

**Definition:** fq\_read.h:49

\_fq\_read

stores a fastq entry

**Definition:** fq\_read.h:37

\_fq\_read::start

int start

**Definition:** fq\_read.h:43

\_fq\_read::packsh

unsigned char packsh[(READ\_MAXLEN+1)/2]

**Definition:** fq\_read.h:48

\_fq\_read::L\_packsh

int L\_packsh

**Definition:** fq\_read.h:52

string\_seq

int string\_seq(Fq\_read \*seq, char \*char\_seq)

writes the fq entry in a string

**Definition:** fq\_read.c:133

\_fq\_read::extended

char extended[READ\_MAXLEN]

**Definition:** fq\_read.h:46

\_fq\_read::Lhalf

int Lhalf

**Definition:** fq\_read.h:45

\_fq\_read::pack

unsigned char pack[(READ\_MAXLEN+1)/2]

**Definition:** fq\_read.h:47

\_fq\_read::line1

char line1[READ\_MAXLEN]

**Definition:** fq\_read.h:38

\_fq\_read::L\_pack

int L\_pack

**Definition:** fq\_read.h:51

get\_fqread

int get\_fqread(Fq\_read \*seq, char \*buffer, int pos1, int pos2, int nline, int read\_len, int filter)

reads fastq line from a buffer

**Definition:** fq\_read.c:55

Fq\_read

struct \_fq\_read Fq\_read

stores a fastq entry


---

Generated on Mon Mar 19 2018 23:42:01 for FastqPuri by  

 1.8.14
